# Columbus Medical College

**Published:** 1847-04

**Authors:** 


ARTICLE V.

### COLUMBUS MEDICAL COLLEGE, OHIO.

By an act of the recent session of the Ohio Legislature, the Willoughby Medical School was removed to Columbus.

The faculty has been re-organized and are now giving their first course of lectures. The faculty is as follows:

J. P. Judkins, M. D., Prof. of Anatomy and Physiology.
R. L. Howard, M. D., Prof. of Surgery.
Rev. F. Merrick, M. D., Prof. of Chemistry.
H. H. Childs, M. D., Prof. of Obstetrics and Diseases of Women and Children.
J. Butterfield, M. D., Prof. of Pathology and practice.
T. R. Spencer, M. D., Prof. of Therapeutics and Mat. Med.
S. M. Smith, M. D., Prof. of Medical Jurisprudence and Insanity.

We are sorry to see the price of a full course of lectures placed so low as fifty-five dollars, being a little less than eight dollars for each ticket.

We had hoped that when the competition between this and the Cleveland school was measurably abated by its removal to Columbus, they would both have fixed their fees at, at least, ten dollars for each Professor's ticket, which is the price almost uniformly adopted by the northern medical colleges.

